# Effects of Transcranial Direct Current Stimulation on Clinical Features of Dizziness and Cortical Activation in a Patient with Vestibular Migraine

**DOI:** 10.3390/brainsci14020187

**Published:** 2024-02-19

**Authors:** Sang Seok Yeo, Chang Ju Kim, Seong Ho Yun, Sung Min Son, Yoon Jae Kim

**Affiliations:** 1 Department of Physical Therapy, College of Health Sciences, Dankook University, Cheonan-si 31116, Republic of Korea; eangbul@hanmail.net; 2 Department of Physical Therapy, College of Health Science, Cheongju University, Cheongju-si 28503, Republic of Korea; 25044398@cju.ac.kr (C.J.K.); ssm0417@hanmail.net (S.M.S.); 3 Department of Health, Graduate School, Dankook University, Cheonan-si 31116, Republic of Korea; yshpt2107@naver.com

**Keywords:** dizziness, functional near-infrared spectroscopy, quantitative electroencephalography, transcranial direct-current stimulation, vestibular migraine

## Abstract

Background: Vestibular migraine (VM) is common migraine that occurs in patients with dizziness. Vestibular rehabilitation for managing VM generally remains unclear. Recently, it has been reported that transcranial direct current stimulation (tDCS) has positive effects in alleviating dizziness. This study investigated the effects of tDCS on dizziness and cortical activation in a patient with VM. Methods: We recruited a male patient aged 31 years with no dizziness. The patient watched a video to induce dizziness using a virtual reality device. The study applied the intervention using tDCS for 4 weeks and measured 4 assessments: functional near-infrared spectroscopy (fNIRS), quantitative electroencephalography (qEEG), dizziness handicap inventory, and visual vertigo analog scale. Results: We showed the activation in the middle temporal gyrus and inferior temporal gyrus (ITG) of the left hemisphere and in the superior temporal gyrus and ITG of the right hemisphere in the pre-intervention. After the intervention, the activation of these areas decreased. In the results of qEEG, excessive activation of C3, P3, and T5 in the left hemisphere and C4 in the right hemisphere before intervention disappeared after the intervention. Conclusions: This study indicated that tDCS-based intervention could be considered a viable approach to treating patients with vestibular dysfunction and dizziness caused by VM.

## 1. Introduction

Dizziness is a condition wherein an individual’s perception of their physical position and direction within a given space is changed through abnormal sensations that are difficult to define [1,2]. It constitutes a major symptom that frequently affects individuals of varying age groups, regardless of gender [1,3]. Dizziness contributes to numerous medical disorders, including presyncope dizziness, psychogenic dizziness, disequilibrium, and vertigo [1]. These can be differentially defined, measured, diagnosed, and treated according to their manifestation [4]. Hence, a step-wise approach must be taken to identify the cause of dizziness accurately [1,4]. Among conditions characterized by dizziness, the most commonly associated with vertigo are benign paroxysmal peripheral vertigo (BPPV), Meniere’s disease, vestibular neuritis, and vestibular migraine (VM) [1].

VM is an altered form of the common migraine condition, potentially affecting more than 7% of patients with dizziness [1,5,6]. Although its specific causes remain unclear, it is known to involve a complex set of factors such as central vascular dysregulation, vestibular dysfunction, and abnormal neuronal activity [1]. Vertigo symptoms may appear due to voluntary movement or postural change [1,7]. Some assessments are based on the diagnostic criteria suggested by Neuhauser & Lempert [8]. It is possible that a general physical examination may yield normal results, while the Romberg test will show positive results for imbalance [1]. As such, vertigo is a phenomenon where an individual perceives their body or the surrounding environment as being abnormally rotated or tilted through a distorted sense of movement, despite the normal movement of the head while moving in a set direction, such as walking forward [1,4].

Therapeutic interventions for vestibular rehabilitation in VM generally remain unclear, and the treatment of patients with acute symptoms mostly relies on drug therapies based on the opinions of a medical specialist [8,9,10,11]. Previous studies have shown that vestibular suppressants could help alleviate symptoms, while others have recommended vestibular training for patients with chronic imbalance and vertigo [3,11]. Recently, studies have indicated the positive effects of vestibular rehabilitation with transcranial direct-current stimulation (tDCS) as an alternative to vestibular training to improve symptoms in patients with chronic dizziness [9,12]. tDCS uses a non-invasive device that creates a microcurrent between the anode and cathode when placed over the cortical areas of the brain [13,14]. The device can affect neural plasticity by controlling excitability and activity in the brain to induce functional improvement [13,14,15,16].

Previous studies on functional neuroimaging methods reported that the symptoms of dizziness and vertigo affected the activation of the cortical vestibular area [17,18,19,20,21,22]. Therefore, the purpose of this study was to confirm the effect of tDCS on clinical features of dizziness and changes in cortical activation in a patient with VM using neuroimaging techniques.

## 2. Methods

### 2.1. Subjects

This study investigated the case of a 31-year-old male patient without a previous diagnosis of dizziness. He was admitted to the neurology department because of sudden syncope during the course of an average day. The initial diagnosis was autonomic dysfunction caused by vagal syncope. The patient was monitored without medication, and no symptomatic improvement was observed for approximately 1 month. He subsequently showed symptoms of nausea, intense headache, vomiting, and abnormal rotation and tilt of the body. For this reason, the patient was referred to another neurology department to ensure an accurate diagnosis. Through the questionnaire, the patient stated that he had experienced migraines of unknown causes irregularly over the past 3 years. The aspects include, as follows: without aura, unpredictable situations, various points on the head, unilateral location, moderate to severe intensity, and lasting more than 10 min. Based on these, a neurologist referred to diagnostic criteria for VM suggested by the Committee for Classification of Vestibular Disorders of the Bárány Society and the Migraine Classification Subcommittee of the International Headache Society (IHS) [23,24], because the patient’s present illness includes intermittent visual aura and photophobia in addition to the previously experienced symptoms. Various methods were applied for differential diagnosis, including the head-up tilt test, the Dix–Hallpike test, the Romberg test, videonystagmography (VNG), and quantitative electroencephalography (qEEG). He stated that the patient had vestibular dysfunction and dizziness caused by a VM of unknown origin. To improve symptoms, the patient was prescribed dizziness and migraine drugs, administered at a set dose twice daily, every day, pre-intervention, and throughout the study. The following drugs were administered daily: Sibelium Cap., Depekote ER Tab. 250 mg, Rabera Tab. 20 mg, Meniase-s Tab., and Grandaxin Tab. Whanin. Almogran Tab. and Mosapri Tab. were administered intermittently in cases of intense headaches.

### 2.2. Transcranial Direct Current Stimulation (tDCS)

For the intervention, we used a tDCS device (ActivaDose II, Caputron, New York, NY, USA) to alter the patient’s neural plasticity by controlling excitability and activity in the brain to cause functional improvement while changes in the clinical features of dizziness were examined. Based on previous studies, the anode and cathode were attached over the right and left hemispheres, respectively, along the parieto-insular vestibular cortex (PIVC) and temporoparietal junction (TPJ) to stimulate the microcurrent [12,13,14]. This placement was determined according to the slightly broader and more intense brainwaves in the left hemisphere with excessive activation in the pre-intervention qEEG results. The patient was instructed to maintain a steady, comfortable, and static standing position without any activity or thought. The intervention was applied for 20 min per session daily for 4 weeks. The tDCS intensity was set at 40.0 (2.0 mA for 20 min).

### 2.3. Functional Near-Infrared Spectroscopy (fNIRS)

In this study, fNIRS (NIRSport2, NIRx Medical Technologies LLC, Berlin, Germany) was used to assess the changes in hemodynamic responses during dizziness according to the intervention period [25]. Based on previous studies, the region of interest (ROI) was defined along the PIVC and TPJ. The design was based on the international standard 10–20 system using the NIRStar software [25,26]. Based on the modified Beer–Lambert law, we acquired values for oxy-hemoglobin (HbO) and total hemoglobin (HbT) following changes in cortical concentration. The patient was in a static standing posture and wore a virtual reality device (Oculus Quest 2, Facebook LLC, Menlo Park, CA, USA). After rest, the patient watched a video (https://youtu.be/4owCynNhpYM) to induce dizziness according to the investigator’s instructions. A single session began with 30 s of preparation, followed by 30 s of video watching and a subsequent 30 s rest period. Three sessions were conducted. At rest, the patient remained comfortable with his eyes closed. Measurements were taken four times at pre-intervention, and 2, 4, and 6 weeks after the intervention. To analyze the collected data, NIRSlab software (ver.2019.04, NIRx Medical Technologies LLC, Glen Head, NY, USA) was used.

### 2.4. Quantitative Electroencephalography (qEEG)

In this study, qEEG (NeuroGuide™, Applied Neuroscience Inc., St. Petersburg, FL, USA, 18 January 2023) was used to assess the changes in brain activity during dizziness according to the intervention period. Based on the international standard 10–20 system, the patient was placed in a comfortable supine position with electrodes attached to the scalp and eyes closed, while following the investigator’s instructions [27]. When stimulation began, the patient kept their eyes closed through a gradual increase in light intensity and velocity. At the end of all stimulations, the patient steadily breathed in a relaxed state according to the instructions of the investigator to complete the assessment. Measurements were taken four times at pre-intervention, and 2, 4, and 6 weeks after the intervention. A device-internal program was used to analyze the collected data. The changes were compared based on the high-beta value associated with stimulation-induced conscious alertness [27]. A level ≥2.0 based on the absolute numerical value of Z was interpreted as a state of excessive activation.

### 2.5. Dizziness Handicap Inventory (DHI)

This study used the DHI to assess the changes in clinical features according to the intervention period. This tool was developed by Jacobson and Newman and is commonly used worldwide with a high level of reliability [28]. It consists of twenty-five questions across the functional, physical, and emotional categories. Each question is rated by marking the level of one’s symptoms as a score of 0 (no), 2 (sometimes), or 4 (yes). A total score ≥10 indicates the need for evaluation by a specialist, while a score of 16–34 indicates a mild state, 36–52 indicates a moderate state, and ≥54 indicates a severe state of dizziness. Prior to the fNIRS and qEEG analyses, the patient self-assessed his clinical features.

### 2.6. Visual Vertigo Analog Scale (VVAS)

This study used the VVAS to assess changes in clinical features according to the intervention period. This tool was developed by Longridge et al. as a rapid self-test to visually assess the level of vertigo [29]. The scale consists of nine questions, each rated by marking the level of pain on a solid-line scale of 0–10 after reading the instructions. The score indicates the absence of pain (0), mild pain (1–3), moderate pain (4–6), and severe pain (7–10). Prior to the fNIRS and qEEG analyses, the patient self-assessed his clinical features.

## 3. Results

### 3.1. Functional Near-Infrared Spectroscopy (fNIRS)

**Figure 1:**
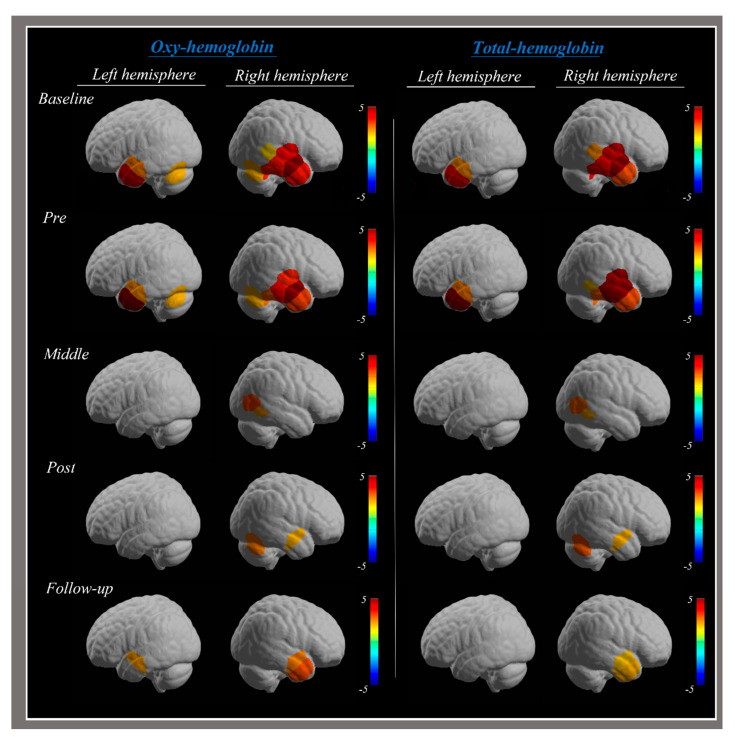
Significant hemodynamic responses in fNIRS analysis. Colors represent hemodynamic responses during patients watching a video that induces dizziness pre-intervention, and 2, 4, and 6 weeks after the intervention.

The post-intervention fNIRS results in this study were as follows: significant HbO and HbT responses were detected in the middle temporal gyrus (MTG) and the inferior temporal gyrus (ITG) of the left hemisphere at pre-intervention, which uniformly decreased at 2 and 4 weeks after the intervention; significant HbO and HbT responses were detected in the superior temporal gyrus (STG), MTG, and ITG of the right hemisphere at pre-intervention, which decreased in the STG and ITG at 2 and 6 weeks after the intervention, respectively (Figure 1; Table 1).

### 3.2. Quantitative Electroencephalography (qEEG)

**Figure 2:**
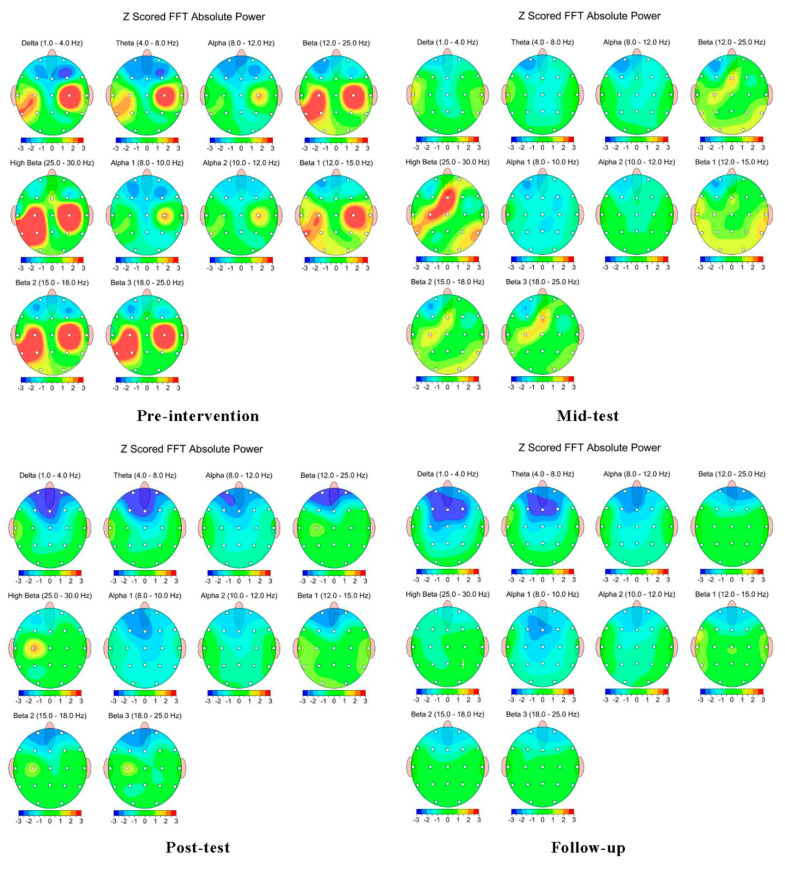
Changes in brain activity related to high beta Z values in qEEG analysis. The red color represents excessive activation in the brain area related to high beta Z values ≥ 2.0. Contrastively, the other colors represent normal activation in the brain area related to high beta Z values < 2.0. Figures indicate analysis at pre-intervention, and 2, 4, and 6 weeks after the intervention, respectively.

The post-intervention qEEG results in this study were as follows: pre-intervention excessive activation observed in C3, P3, and T5 in the left hemisphere disappeared in P3 at 2 weeks after the intervention, in T5 at 4 weeks after the intervention, and in C3 at 6 weeks after the intervention. Excessive activation of C4 in the right hemisphere pre-intervention disappeared 2 weeks after the intervention. After 6 weeks, the level of activation was reduced from 6.05 to −0.81 in C3, from 7.89 to −0.48 in P3, from 8.95 to −0.28 in T5, and from 8.94 to 0.32 in C4 (Figure 2; Table 2).

### 3.3. Dizziness Handicap Inventory (DHI)

The post-intervention DHI results in this study were as follows: the score was 74 pre-intervention, which declined to 56 at 2 weeks, 42 at 4 weeks, and 30 at 6 weeks after the intervention, with an overall decrease of 44 after 6 weeks. The decrease in scores after 6 weeks per category was 14 for functional, 12 for physical, and 18 for emotional (Table 3).

### 3.4. Visual Vertigo Analog Scale (VVAS)

The post-intervention VVAS results in this study were as follows: pre-intervention, two items were scored severe, and seven items were scored moderate. Scores improved to moderate for six items and mild for three items at 2 weeks, moderate for four items and mild for five items at 4 weeks, and moderate for one item and mild for eight items at 6 weeks after the intervention (Table 3).

## 4. Discussion

This study aimed to verify the improvements in the clinical features of a patient with vestibular dysfunction and dizziness caused by VM following tDCS intervention. For relevant assessments, hemodynamic responses were analyzed using fNIRS, brain activity changes were examined using qEEG, and perceived pain levels were compared using DHI and VVAS. The intervention period was 4 weeks from the onset of the study, and follow-up monitoring was performed for 2 subsequent weeks.

The fNIRS results indicated that the significant HbO and HbT responses in the MTG and ITG of the left hemisphere before the intervention uniformly decreased after the intervention, and the significant HbO and HbT responses in the STG, MTG, and ITG of the right hemisphere decreased after the intervention, except in the MTG. The reduced hemodynamic responses in the STG and MTG related to dizziness can be taken to indicate improved clinical features after the intervention period [17,18,19,20,21,22]. However, the redetected significant response in the MTG of the left hemisphere 6 weeks after the intervention and the continuous significant response in the MTG of the right hemisphere during the intervention period were in line with a previous study reporting increased blood flow in chronic dizziness patients [30]. The patient in this study was given the intervention after ensuring an accurate diagnosis one month after the onset of initial symptoms; hence, this cannot be regarded as treatment of an acute case. During the 4 weeks of intervention, significant responses were continuously detected in the ITG of the right hemisphere, which could be a feature induced by the tDCS device that stimulates the intended areas and adjacent due to the size of the sponge with the anode attachment [13,14].

The qEEG results indicated that the excessive activation of C3, P3, T5, and C4 in both hemispheres before the intervention was replaced by stabilized levels within the normal range in all areas that exhibited high beta Z values after the intervention to indicate conscious alertness. In particular, sudden changes 2 weeks after the intervention correlated with previous studies reporting improved clinical features of dizziness after vestibular rehabilitation with tDCS [9,12]. This may alternately be interpreted as a functional improvement due to changes in neural plasticity through the control of brain excitability and activity due to microcurrent stimulations [13,14,15,16]. Additionally, comparing the fNIRS and qEEG results together shows that adjusted patterns of brain activity, with changes in neural plasticity, could have led to hemodynamic responses, which influenced the symptomatic improvement of dizziness. In a previous study, robot-assisted gait training with concurrent fNIRS and qEEG analyses was suggested as a promising method of intervention for neurorehabilitation in stroke patients with gait or balance disorders [31]. This intervention could improve gait recovery through potential changes in neural plasticity. In another previous study, a method to differentiate patients with Parkinson’s disease from those with other neurodegenerative disorders was suggested in which cerebral activities were recorded and overall movements were analyzed [32]. In line with these findings, this study may also offer an integrated interpretation of fNIRS and qEEG results. However, the patient in this study was already taking medication, and owing to ethical considerations, the overlapping effect of the respective drugs could not be ruled out.

The DHI results indicated that the severe level of dizziness handicap before the intervention was reduced to a mild level across all functional, physical, and emotional categories. The VVAS results indicated that visual vertigo of severe and moderate levels before the intervention was reduced to moderate and mild levels (for one item and eight items, respectively) following the 6-week intervention period. In a previous study that concurrently applied the DHI and VVAS, the two methods were shown to be useful in identifying the level of dizziness that could affect the quality of life of patients with vestibular dysfunction [28]. Based on this, the results of this study could be used to indicate an improvement in the clinical features of dizziness after the intervention period, whereby the changes in the DHI and VVAS were positively correlated.

In conclusion, the tDCS-based intervention in this study changed the neural plasticity involved in cerebral activities to restore a stable state through microcurrent stimulation, as indicated by the measurements of hemodynamic responses. Based on these findings, this intervention is suggested as an effective vestibular rehabilitation method that can ensure the improvement of clinical symptoms for patients with vestibular dysfunction and dizziness. The limitations of this study are as follows: First, it was difficult to objectively verify the interventional effects as only one patient participated in this study. Second, because the patient was on previously prescribed medications, the overlapping effects of the respective drugs could not be ruled out, owing to ethical considerations. Third, as the intervention was applied 1 month after the onset of vestibular dysfunction and dizziness, the compensatory effects from other organs could not be ruled out. Thus, a follow-up study should be conducted to resolve these limitations and establish specific and detailed directions. Based on the findings of this study, despite a few limitations, tDCS-based intervention is suggested as an effective method for improving the clinical features of patients with vestibular dysfunction and dizziness caused by VM.

## 5. Conclusions

This study aimed to validate the effects of tDCS-based intervention on enhancing clinical features and cortical activation in a patient with vestibular dysfunction and dizziness caused by VM. Following the intervention, the clinical features of dizziness were significantly improved, leading to reduced scores in DHI and VVAS. Additionally, a considerable change in cortical activation was observed, resulting in levels comparable to a normal person in fNIRS and qEEG. Based on these findings, we suggest that tDCS-based intervention could be considered a viable approach to treat patients with vestibular dysfunction and dizziness caused by VM.

## Figures and Tables

**Table 1:** Significant channels of HbO and HbT in fNIRS analysis.

Session	Brain Region (Brodmann Area)		HbO	HbT
Baseline	Rt	Superior Temporal Gyrus (BA 22)	25	25
		Middle Temporal Gyrus (BA 21)	6, 24, 26, 33	6, 24, 26, 33
		Inferior Temporal Gyrus (BA 20)	20, 35	35
	Lt	Middle Temporal Gyrus (BA 21)	23, 34	23, 34
		Inferior Temporal Gyrus (BA 20)	17	-
Pre-test	Rt	Superior Temporal Gyrus (BA 22)	25	25
		Middle Temporal Gyrus (BA 21)	24, 26, 33	24, 26, 33
		Inferior Temporal Gyrus (BA 20)	20, 35	35
	Lt	Middle Temporal Gyrus (BA 21)	23, 34	23, 34
		Inferior Temporal Gyrus (BA 20)	17	17
Mid-test	Rt	Middle Temporal Gyrus (BA 21)	18	18
		Inferior Temporal Gyrus (BA 20)	19	19
Post-test	Rt	Middle Temporal Gyrus (BA 21)	26	26
		Inferior Temporal Gyrus (BA 20)	20	20
Follow-up	Rt	Middle Temporal Gyrus (BA 21)	26, 33	26, 33
	Lt	Middle Temporal Gyrus (BA 21)	23	-

HbO: oxyhemoglobin, HbT: total hemoglobin, BA: Brodmann area, fNIRS: functional near-infrared spectroscopy.

**Table 2:** Changes in brain activity related to high beta Z values in qEEG analysis.

		Pre-Test	Mid-Test	Post-Test	Follow-Up
Rt	FP2	0.24	1.13	−1.11	−1.32
	F4	−0.99	−0.08	0.05	−0.59
	C4	8.94	−0.48	−0.20	0.32
	P4	1.49	1.03	−0.37	0.52
	O2	0.45	1.37	−0.08	0.31
	F8	−0.49	−0.23	−0.59	−0.48
	T4	−0.05	−0.01	−0.40	0.23
	T6	0.17	2.30	−0.24	−0.30
Lt	FP1	−0.87	−0.73	−1.42	−1.09
	F3	−0.74	−0.68	−0.88	−0.58
	C3	6.05	2.76	2.18	−0.81
	P3	7.89	−0.08	−0.55	−0.48
	O1	0.75	0.14	−0.30	0.02
	F7	0.04	0.04	−0.77	−0.76
	T3	−0.43	0.50	−0.45	−0.17
	T5	8.95	2.28	0.03	−0.28

qEEG: quantitative electroencephalography.

**Table 3:** Changes in the clinical features of dizziness.

		Pre-Test	Mid-Test	Post-Test	Follow-Up
DHI	Functional	26	22	18	12
	Physical	24	18	14	12
	Emotional	24	16	10	6
	Total	74	56	42	30
VVAS	Mild	0	3	5	8
	Moderate	7	6	4	1
	Severe	2	0	0	0
	High	8	6	5	4
	Low	6	3	2	2

DHI: dizziness handicap inventory, VVAS: visual vertigo analog scale.

## Data Availability

The datasets generated and/or analyzed during the current study are not publicly available due to the privacy of the patient, but are available from the corresponding author upon reasonable request.
